# Anti-inflammatory effects of the combined extracts of *Achyranthes japonica nakai* and *Aralia continentalis kitagawa in vitro* and *in vivo*

**DOI:** 10.1016/j.dib.2019.104088

**Published:** 2019-06-03

**Authors:** Young Min Woo, Ok Ju Kim, Eun Sol Jo, Su Jin Kim, Young-Ho Lee, Mee Young Ahn, Sang-Hyeon Lee, Jong-Myung Ha, Andre Kim

**Affiliations:** a Department of Natural Science Institute, Silla University, 46958, Busan, Republic of Korea; b Pharmaceutical Engineering, Division of Bioindustry, College of Medical and Life Sciences, Silla University, 46958, Busan, Republic of Korea; c Department of Natural Science Institute, Medi&Bio Co., Ltd., Research & Development Center, 01811, Seoul, Republic of Korea; d Protein Structure Group, Korea Basic Science Institute, 28119, Chungcheongbuk-do, Republic of Korea

## Abstract

This study investigated the anti-inflammatory effects of mixed extracts of *Achyranthes japonica Nakai* (AJ) and *Aralia continentalis Kitagawa* (AC) (ratios of 1:2, 1:3, 1:5, 2:1, 3:1 and 5:1) on RAW264.7 macrophages and evaluated the anti-inflammatory effects of the mixed extracts of AJ and AC by measuring IL-1*β*, IL-6, and TNF*α* using the ELISA kit assay. In particular, the formation of nitric oxide (NO) was found to decrease in the group treated with the combined extracts of AJ and AC at all ratios. In particular, extracts of ratio of 2:1 (AJ:AC) deceased the formation of NO level that is approximately 60% of the group treated with only lipopolysaccharide (LPS). Also, extracts of ratio of 2:1 (AJ:AC) reduced the production of IL-1*β*, IL-6, TNF*α* and PGE2 with statistical significance. Volunteers over the age of 50 who complain of discomfort in knee joints were selected as the experimental subjects. The subjects took daily administration of 2000 mg of the combined extracts of ratio of 2:1 (AJ:AC) for 12 weeks. A survey (VAS (Visual Analog Scale), WOMAC (Western Ontario and McMaster Universities Osteoarthritis Index)) was conducted after the 12 weeks of oral administration. The experimental group showed the change between each visit and baseline time compared with the control group. In the intention-to-treat (ITT) analysis, VAS score and WOMAC stiffness score decreased significantly. And the WOMAC total score and function score tended to decrease. In the per-protocol (PP) analysis, the WOMAC stiffness score was significantly decreased and the VAS and WOMAC total and function scores were decreased. There was no significant difference in all parameters of ITT and PP in radiological examinations.

**Table:** Specifications table

Subject area	*biochemistry*
More specific subject area	*Food chemistry*
Type of data	*Table, graph*
How data was acquired	*ELISA, survey(Visual Analog Scale, Western Ontario and McMaster Universities Osteoarthritis Index)*
Data format	*Analyzed*
Experimental factors	*Function and bone/joint health*
Experimental features	*Volunteers who complain of discomfort in knee joints comparing the change between each visit and the baseline time point, the experimental group showed the following effects compared with the control group.*
Data source location	*Silla University*
Data accessibility	*Data are available within this article*

**Table:** 

**Value of the data** • The combined extracts of Achyranthes japonica Nakai and Aralia continentalis Kitagawa prevent the manifestation of inflammation by restraining the creation of nitric oxide and inflammatory cytokine through control of the activation of macrophage. • The combined extracts of AJ and AC improve immunity and reduce hypersensitive immune reaction. • The combined extracts of AJ and AC help to improve immunity function and bone/joint health.

## 1 Data

**Fig. 1:**
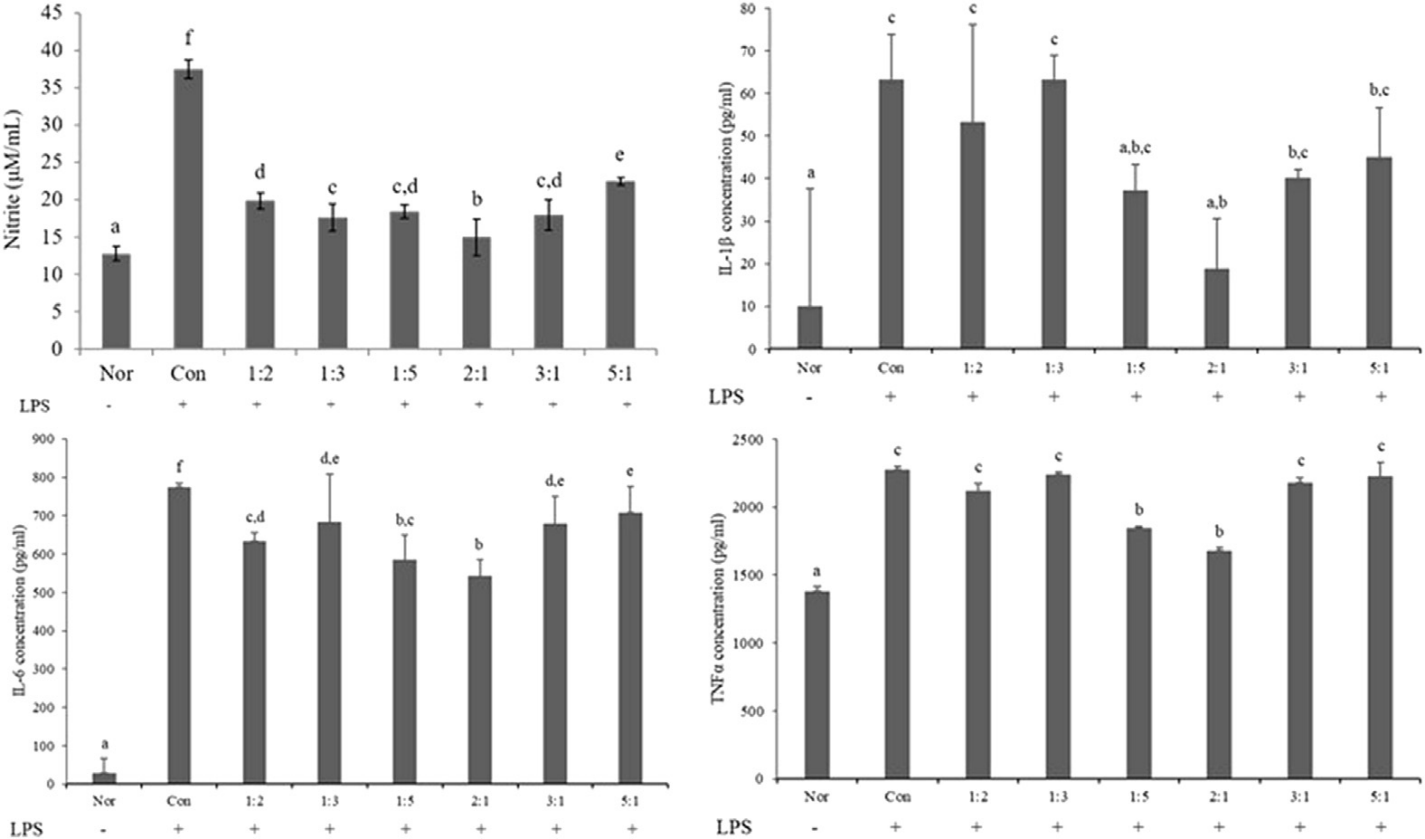
Effects of the combined extracts of AJ and AC on nitric oxide, IL-1β, IL-6, and TNFα generation in LPS-induced RAW264.7 macrophage cells.

The result of the production of nitric oxide, IL-1β, IL-6, and TNFα, the inflammatory cytokines produced by RAW264.7 macrophages and induced by LPS stimulation in combined extracts of *Achyranthes japonica Nakai* (AJ) and *Aralia continentalis Kitagawa* (AC), are depicted in Fig. 1. In the RAW264.7 cells treated with lipopolysaccharide(LPS), the formation of nitric oxide (NO) was found to decrease in the group treated with the combined extracts of AJ and AC at all ratios. In particular, at the ratio of 2:1 (AJ:AC), the formation of NO was found to fall to the level that is approximately 60% of the group treated with only LPS.

In the measurement of IL-1β, the group treated with only LPS showed 63.26 pg/ml, which was larger than the control group that was not treated with LPS (10.12 pg/ml). The IL-1β content in ratios of 2:1, 1:5, and 3:1 were 18.81, 37.35, and 40.14 pg/ml, respectively, indicating restricted creation. In particular, the creation of IL-1β was largely restricted in the case of the 2:1 ratio, in which the IL-1β content was approximately 70% of the control group. In the measurement of IL-6, the group treated with only LPS showed a significantly higher value of 774.49 pg/ml, compared to the IL-6 level of 678.98, 633.52, and 584.76 pg/ml at ratios of 3:1, 1:2, and 1:5, respectively. In particular, the IL-6 content was 542.68 pg/ml at the ratio of 2:1, which is approximately 30% that of the control group. The control group showed the TNFα content of 2275.68 pg/ml, which is 39% higher than that of the normal group (1379.47 pg/ml). However, TNFα was 1847.02 pg/ml and 1679.11 pg/ml at the ratios of 1:5 and 2:1, respectively, which is a decrease of 19% and 26%, respectively.

**Fig. 2:**
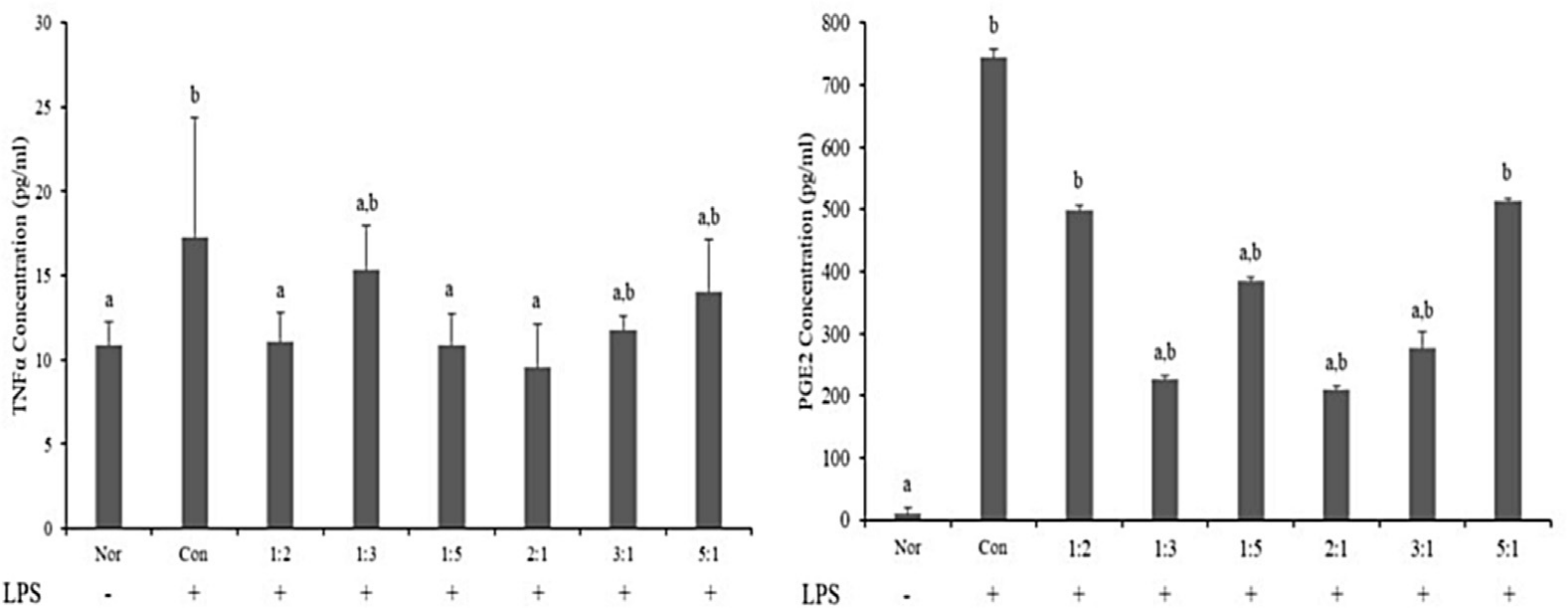
Effects of the combined extracts of AJ and AC on TNFα and PGE2 generation in SD-rat serum.

Fig. 2 shows the measurement of TNFα and PGE_2_ in the serum obtained from the SD-rat after two-week oral administration of the combined extracts of AJ and AC. In the measurement of TNFα, the group treated with only LPS showed a more significant increase of 17.25 pg/ml than the normal group. The creation of TNFα was significantly restricted at the ratios of 2:1, 1:5, and 1:2 (AJ:AC), with TNFα contents of 9.60, 10.85, and 11.08 pg/ml, respectively. In particular, the group with the ratio of 2:1 showed a 44% decrease compared with the control group. In the measurement of PGE_2_, the control group showed 743.85 pg/ml, indicating a larger increase than the normal group (9.60 pg/ml). The creation of PGE_2_ was significantly restricted at ratios of 2:1, 1:3, and 3:1, with PGE_2_ contents of 208.86, 226.61, and 276.45 pg/ml, respectively.

Table 1 shows the results of the analysis of the nutritive components of the combined extracts of AJ and AC that were manufactured at the ratio of 2:1 (AJ:AC) based on the in vitro outcome above. Table 2 shows the results of the indicator content analysis.

**Table 1:** Nutritional analysis results.

Test items	Contents
Energy (Kcal/100 g)	353.36
Carbohydrate (%)	77.92
Protein (%)	5.11
Fat (%)	2.36
Sodium (mg/100 g)	117.22
Ash content (g/100 g)	8.43
Water content (g/100 g)	6.18

**Table 2:** Analysis of indicator material content.

Lot. No.	AJ (mg/g)	AC (mg/g)
1	5.73	2.33
2	4.87	2.34
3	5.13	2.62
4	5.10	2.61
5	5.19	2.66
6	5.14	2.62
7	5.14	2.64
8	5.18	2.64
Average	5.19	2.56

Table 3 compares the change of each visit point with respect to the base point. Table 4 shows the results of calibrating the questionnaire survey results based on calorie intake and activity level, which showed significant differences for 12 weeks.

**Table 3:** Changes of VAS and WOMAC scores for 12 weeks^a^.

Variables	Placebo	AA	P-value^b^		
			Group	Week	Group*week
VAS score (mm)					
Week 4	6.00 ± 2.02	−1.78 ± 2.05			
Week 8	3.76 ± 2.02	1.05 ± 2.11			
Week 12	5.03 ± 2.02	−2.81 ± 2.14	0.028	0.440	0.038
P-value^c^	0.028	0.236			
WOMAC scoreTotal					
Total					
Week 4	−0.67 ± 2.30	−5.31 ± 2.32			
Week 8	−6.15 ± 2.30	−11.26 ± 2.38			
Week 12	−4.33 ± 2.30	−12.80 ± 2.40	0.077	<0.001	0.087
P-value^c^	0.060	<0.001			
Pain					
Week 4	0.03 ± 0.52	−0.87 ± 0.52			
Week 8	−1.03 ± 0.52	−1.91 ± 0.53			
Week 12	−0.94 ± 0.52	−1.56 ± 0.54	0.299	<0.001	0.570
P-value^c^	0.068	0.004			
StiffnessTotal					
Week 4	0.00 ± 0.23	−0.47 ± 0.23			
Week 8	−0.33 ± 0.23	−1.30 ± 0.24			
Week 12	−0.06 ± 0.23	−1.36 ± 0.24	0.006	<0.001	0.002
P-value^c^	0.807	<0.001			
FunctionTotal					
Week 4	−0.70 ± 1.70	−3.97 ± 1.72			
Week 8	−4.79 ± 1.70	−8.05 ± 1.76			
Week 12	−3.33 ± 1.70	−9.88 ± 1.78	0.084	<0.001	0.077
P-value^c^	0.054	<0.001			

a LSmean ± SE (all such values). AA, *Achyranthes japonica nakai* and *Aralia continentalis Kitagawa*; VAS, visual analogue scale; WOMAC, Western Ontario McMaster University Osteoarthritis Index.

b Linear mixed-effect model was used to analyze the effects of group, week and group*week.

c Linear mixed-effect model was used to analyze the difference within each group.

**Table 4:** Changes of VAS and WOMAC scores for 12 weeks^a^.

Variables	Placebo	AA	P-value^b^		
			Group	Week	Group*week
VAS score (mm)					
Week 4	6.11 ± 2.06	−1.74 ± 2.08			
Week 8	3.75 ± 2.04	0.87 ± 2.14			
Week 12	4.91 ± 2.04	−2.64 ± 2.15	0.034	0.445	0.041
P-value^c^	0.029	0.264			
WOMAC score					
Total					
Week 4	−0.66 ± 2.34	−5.53 ± 2.36			
Week 8	−6.01 ± 2.32	−11.10 ± 2.41			
Week 12	−4.05 ± 2.32	−13.09 ± 2.42	0.067	<0.001	0.084
P-value^c^	0.060	<0.001			
Pain					
Week 4	0.09 ± 0.52	−0.97 ± 0.53			
Week 8	−0.96 ± 0.52	−1.92 ± 0.54			
Week 12	−0.85 ± 0.52	−1.63 ± 0.54	0.211	<0.001	0.619
P-value^c^	0.072	0.005			
Stiffness					
Week 4	−0.03 ± 0.23	−0.47 ± 0.24			
Week 8	−0.33 ± 0.23	−1.26 ± 0.24			
Week 12	−0.03 ± 0.23	−1.40 ± 0.24	0.007	<0.001	0.002
P-value^c^	0.830	<0.001			
Function					
Week 4	−0.72 ± 1.73	−4.08 ± 1.75			
Week 8	−4.72 ± 1.72	−7.93 ± 1.79			
Week 12	−3.17 ± 1.72	−10.06 ± 1.80	0.079	<0.001	0.072
P-value^c^	0.056	<0.001			

a LSmean ± SE (all such values). AA, *Achyranthes japonica nakai* and *Aralia continentalis Kitagawa*; VAS, visual analogue scale; WOMAC, Western Ontario McMaster University Osteoarthritis Index.

b Linear mixed-effect model adjusted with energy intake and physical activity for 12 weeks was used to analyze the effects of group, week and group*week.

c Linear mixed-effect model adjusted with energy intake and physical activity for 12 weeks was used to analyze the difference within each group.

Men and women over the age of 50 who complain of discomfort in knee joints were selected as the experimental subjects. The subjects took daily administration of 2000 mg of the combined extracts of AJ and AC for 12 weeks. A survey (VAS (Visual Analog Scale), WOMAC (Western Ontario and McMaster Universities Osteoarthritis Index)) was conducted after the 12 weeks of oral administration. Comparing the change between each visit and the baseline time point, the experimental group showed the following effects compared with the control group. In the case of intention-to-treat (ITT), the VAS score (P = 0.038) and WOMAC stiffness score (P = 0.002) significantly decreased and the WOMAC total score (P = 0.087) and function score (P = 0.077) showed a downward trend. The questionnaire survey results were calibrated using the intake of calories and activity level during the 12 weeks and showed a significant difference among the subjects. After the calibration, the VAS score (P = 0.041) and WOMAC stiffness score (P = 0.002) significantly decreased and the WOMAC total score (P = 0.084) and function score (P = 0.072) showed a downward trend, indicating a similar result to the non-calibrated outcome. In case of the per-protocol (PP) analysis, the WOMAC stiffness score (P = 0.003) significantly decreased and the VAS score (P = 0.076), WOMAC total score (P = 0.089), and function score (P = 0.078) showed a downward trend. The WOMAC stiffness score (P = 0.003) significantly decreased after the calibration of the survey results based on the “BMI at the baseline and intake of calories and activity level during the 12 weeks.” The VAS score (P = 0.084), WOMAC total score (P = 0.085), and function score (P = 0.073) showed a downward trend, indicating a similar result to the non-calibrated outcome. No significant between-group difference was observed in all markers of ITT and PP.

## 2 Experimental design, materials and methods

The concentrations of NO produced in the RAW264.7 cell supernatants were measured by quantification using the griess method to identify the immunity boosting ability of the combined extracts of AJ and AC. The RAW264.7 cells were dispensed into 60 mm dishes at a concentration of 1.5 × 10^5^ cells/dish; after 24 hours, they were treated with 1 μg/mL LPS and then cultured for 24 hours. The combined extracts of AJ and AC were treated at 400 μg/mL, the maximum concentration without toxicity according to the results of the CCK assay; after 24 hours, the experiment was conducted using supernatants. The supernatant of cells without LPS treatment was used for the positive control group and the supernatant of cells treated with only 1 μg/mL LPS was used for the negative control group. After a 100 μl supernatant mixed with the same amount of the griess reagent was kept at room temperature for 15 minutes for a reaction, its absorbance was measured at 540 nm using an enzyme linked immunosorbent assay (ELISA) plate reader. Here, the standard curve was drawn using sodium nitrite.

In order to measure the effects of the combined extracts of AJ and AC on the volumes of IL-1β, IL-6, and TNFα produced by LPS stimulation, RAW264.7 macrophages were dispensed into 60 mm dishes at a concentration of 1.5 × 10^6^ cells/dish and then cultured for 24 hours. After inflammations were induced for 24 hours by applying LPS (1 μg/mL), each of the 6 different samples of the combined extracts of AJ and AC was processed. After 24 hours, the cell culture fluids were collected and the supernatants that had undergone the process of centrifugation were preserved at −20 °C for use as samples. Further, the levels of cytokine production were measured using an ELISA kit (R&D Systems, Minneapolis, MN, USA) in accordance with its testing guidelines, and the absorbance was measured using the ELISA reader [1], [2].

Six-week-old white male rats of the Sprague-Dawley strain (Samtako, Seoul, Korea) were used as experimental animals after being stabilized for one week. The rats were divided into eight groups, and each group (control, normal, 1:2, 1:3, 1:5, 2:1, 3:1, and 5:1 [AJ:AC]) received oral administration of the combined extracts of AJ and AC for two weeks. After the two-week administration, blood was collected, from which the serum was obtained through the centrifugation for 15 minutes under the condition of 3000 rpm, 4 °C. The serum's TNFα and PGE_2_ production was measured using an ELISA kit (R&D Systems, Minneapolis, MN, USA) in accordance with its testing guidelines, and the absorbance was measured using the ELISA reader [3].

The nutritive components of the specimen fabricated using a ratio of 2:1 through the manufacturing process were analyzed by the Korea Health Supplements Institute. The human application study was conducted by the Jecheon Oriental Medicine Hospital affiliated with Semyung University and the Department of Food Science and Technology at the Seoul National University of Science and Technology.
